# Mobilizing Action Toward Community Health (MATCH): Metrics, Incentives, and Partnerships for Population Health

**Published:** 2010-06-15

**Authors:** David A. Kindig, Bridget C. Booske, Patrick L. Remington

**Affiliations:** University of Wisconsin School of Medicine and Public Health, Population Health Institute; University of Wisconsin, Madison, Wisconsin; University of Wisconsin, Madison, Wisconsin

How are we doing — and how can we do better? These are perhaps the most basic questions a community can ask regarding the health of its residents. Yet communities have not been given the necessary tools to answer these questions with validated, consistent measures, evidence-based policies and practices, and incentives for improvement.

In response to this need and with funding from the Robert Wood Johnson Foundation, we initiated a project called Mobilizing Action Toward Community Health (MATCH) at the University of Wisconsin-Madison Population Health Institute (1). We created a logic model (Figure) that guides our work and demonstrates the principal activities of 1) producing county health rankings in all 50 states, 2) examining partnerships and organizational models to increase involvement and accountability for population health improvement, and 3) developing incentive models to encourage and reward communities that implement evidence-based programs and policies that improve population health.

**Figure:**
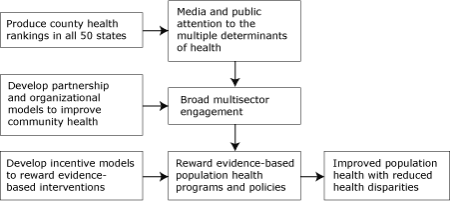
The Mobilizing Action Toward Community Health (MATCH) logic model. This model shows how incentives can be used to improve population health and reduce health disparities.

We believe that together these efforts will increase awareness of the multiple determinants of health, promote engagement by a more diverse group of stakeholders, and stimulate development of models that promote evidence-based programs and policies — eventually leading to improved health outcomes and reduced health disparities.

The most visible product of this effort so far is the county health rankings (2) released in early 2010. Several other components of our project, based in part on a proposed “pay-for-population-health” performance system advanced in 2006 (3), are aimed at understanding how we might best support population health improvement at the community level. To that end, we commissioned 24 essays to critique the assumptions underlying such a system and to suggest approaches for overcoming potential barriers to its implementation. We worked with these authors, MATCH and Robert Wood Johnson Foundation staff, and several guests in a 2-day meeting in late 2009 in Madison to discuss the essays and develop an agenda for future practice and research activities for improving population health.

**Figure:**
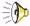
Why Metrics Matter (MP3–3Mb) Listen to an interview with David Kindig, MD, PhD, professor emeritus at the University of Wisconsin Population Health Institute and co-principal investigator on the MATCH initiative. Dr Kindig briefly explains why metrics matter and comments on the changing landscape of data collection.

00:00:00 Fran Kritz I’m Fran Kritz, editor of the Robert Wood Johnson Foundation public health page. Mobilizing Action Toward Community Health, better known as MATCH, is a groundbreaking initiative led by the University of Wisconsin Population Health Institute and funded by the Robert Wood Johnson Foundation.
00:00:16 Kritz The goal of the project is to serve as a nationwide call to action for improving health. MATCH rolled out its efforts last February with the county health rankings.
00:00:26 Kritz We’re talking today with Dr David Kindig, who is professor emeritus at the Population Health Institute at the University of Wisconsin and also the co-principal investigator for the MATCH project.
00:00:37 Kritz Dr Kindig, welcome. 
Dr David Kindig Thank you for talking to me.
00:00:41 Kritz We are talking today because there’s a new project and that is the new issue of the CDC’s online journal Preventing Chronic Disease, includes several essays that were commissioned by the MATCH project and the Robert Wood Johnson Foundation.
00:00:55 Kritz Can you highlight some of the key research among the essays in the journal?
Kindig Sure. We were really privileged to have a number of the nation’s experts on metrics contribute essays to the project and coming out next week in the journal.
00:01:13 Kindig As you mention before, a large part of our MATCH project beyond the county health rankings themselves is essentially to think through and provide advice about taking action and really improving population health.
00:01:26 Kindig And there’s an old saying that you can’t manage what you can’t measure and so this first issue focuses on the measurement piece: ways of thinking about outcomes, about disparities, about the different determinants, and so there’s papers on medical care metrics and socioeconomic metrics and environmental metrics.
00:01:51 Kindig So each of the essay writers has up-to-date, current thinking on current and possibly future metrics.
00:01:59 Kindig In addition, there’s a couple of inter, there’s a couple of commentaries that start it out and particularly Linda Bilhiemer from the CDC has a nice piece about how do we evaluate the metrics in terms of their usefulness for improving population health.
00:02:15 Kritz How might some of the metrics be used in future national county health rankings projects and by individual communities?
00:2:23 Kindig Sure, well, um, as you know the county health rankings will be done annually and we are hoping to keep the outcomes measures the same, so we can track the overall health of counties over time in a valid way.
00:02:39 Kindig But as, hopefully as some of your listeners know, we also rank counties on their determinants of health. So metrics about medical care, social factors, environmental factors, and those we will change over time as new measures become available, particularly at the county level. Often we have trouble getting robust best measures for all kinds of small counties. So we will do that.
00:03:03 Kindig But also, this goes beyond the rankings. I mean, individual states and communities may want to look at things their own way. Some areas may have better data so they can do more than we can do for every county. I’m hopeful that Healthy People 2020 and a lot of the state 2020 projects will look to these as possibilities for useful, for ways they can enhance their own metrics. 
00:03:31 Kritz And health disparity is a key focus, I know. Using metrics to capture health disparity is a focus in some of the essays. Why is that data so pivotal?
00:03:40 Kindig Well, you know, we talk about two goals of the nation: improving our overall health and reducing disparities. And I think frankly we spend a lot more time talking about the overall health, in metrics at least, and less time on actually careful metrics for health disparities, particularly for overall, um, overall, um, disparity outcomes like mortality, quality of life, and healthy days. 
00:04:08 Kindig And even in the county rankings they’re a disparity measure in themselves because they compare geographies. But we don’t in that exercise explicitly look within counties with disparities issues like race, socioeconomic status or gender. 
00:04:26 Kindig And so there’s a couple of, many of the essay writers talk about that and there’s some overall essays about the critical importance of tracking disparities with exactly the same rigor and vigor that we track, say, overall population health means. 
00:04:43 Kritz Having the data of course requires gathering much of it from individuals, and up until recently the way that that data was typically gathered was through surveys done by landline telephones. Now of course people use landline telephones and cell phones, social media, e-mail for communicating. How does that change how the data is gathered?
00:05:04 Kindig Ya, well that’s a really important question. A number of the essays touch on it. The one on behavior by Mokdad and Remington specifically talks about that because a lot of the data we have on behaviors like smoking rates, and um, obesity rates and some of those things actually come from phone surveys, a lot of them the Behavioral Risk Factor Surveillance System from the CDC.
00:05:28 Kindig And some of that data is becoming problematic because of cell phones and other kinds of things. So they point out, and in addition to enhancing those systems and making them as good as they can be, we’re going to have to look at data from other institutional settings, like what you can get from medical records, and health care providers, or in schools, or from employers. All those databases, as well as sort of Internet-based surveys.
00:05:58 Kindig So there will be, there undoubtedly will be advances in the future on how we learn about these things and measure them.
00:06:08 Kritz Doctor David Kindig, thank you so much for discussing the upcoming issue of Preventing Chronic Disease.
Kindig Thanks so much, it was a pleasure talking to you.
Kritz I’m Fran Kritz for the Robert Wood Johnson Foundation.

In this issue of *Preventing Chronic Disease*, we present the 7 essays on population health metrics (4-10), introduced by 2 commentaries (11,12). These essays describe the types of tools that can be used to measure and monitor the health of populations and are the first of 3 sets of essays to appear in this and the next 2 issues.

The next set of essays will describe incentives that can be used to promote programs and policies that improve population health, and the role for population health partnerships in these efforts. The final set will summarize the discussion of the 2009 meeting and outline cross-cutting themes and priorities for research and practice in population health improvement. We hope that the essays will stimulate discussion and mobilize action that improves population health outcomes in the coming decade.
